# Supplementary material for: Occult Vertebral Fracture (OVF) in Patients Who Underwent Hepatectomy for Colorectal Liver Metastasis: Strong Association with Oncological Outcomes
Source: Cancers (Basel). 2023 Nov 22;15(23):5513. doi: 10.3390/cancers15235513 (PMC10705116; doi:10.3390/cancers15235513)

Supplement Figure S1. Kaplan–Meier curve for cancer specific survival after hepatic resection for colorectal liver metastases according to the treatment of recurrence. (A) Non-occult vertebral fracture (OVF) group (B) occult vertebral fracture (OVF) group

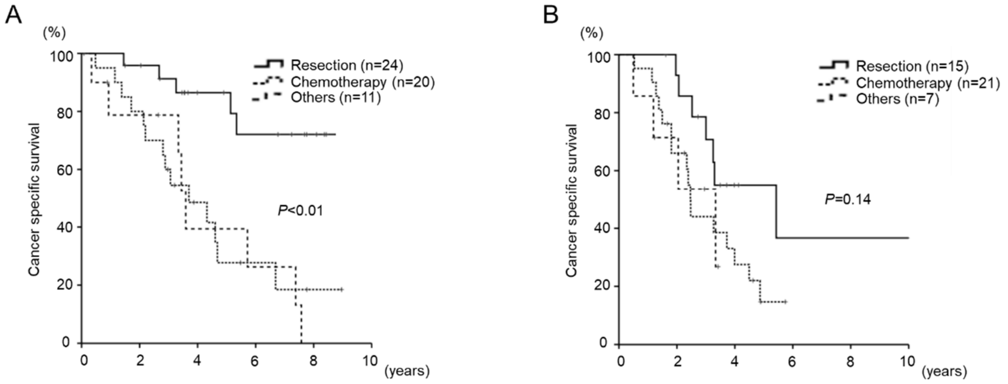

Supplement: Supplementary file 1 [file cancers-15-05513-s001.zip › cancers-2662506-supplementary.pdf]
